# Nitrogen Addition Significantly Affects Forest Litter Decomposition under High Levels of Ambient Nitrogen Deposition

**DOI:** 10.1371/journal.pone.0088752

**Published:** 2014-02-14

**Authors:** Li-hua Tu, Hong-ling Hu, Gang Chen, Yong Peng, Yin-long Xiao, Ting-xing Hu, Jian Zhang, Xian-wei Li, Li Liu, Yi Tang

**Affiliations:** 1 College of Forestry, Sichuan Agricultural University, Ya’an, Sichuan, P.R. China; 2 Maize Research Institute, Sichuan Agricultural University Chengdu Campus, Wenjiang District, Chengdu, Sichuan, P.R. China; 3 Personnel Department, Sichuan Agricultural University, Ya’an, Sichuan, P.R. China; 4 College of Horticulture, Sichuan Agricultural University, Ya’an, Sichuan, P.R. China; Tennessee State University, United States of America

## Abstract

**Background:**

Forest litter decomposition is a major component of the global carbon (C) budget, and is greatly affected by the atmospheric nitrogen (N) deposition observed globally. However, the effects of N addition on forest litter decomposition, in ecosystems receiving increasingly higher levels of ambient N deposition, are poorly understood.

**Methodology/Principal Findings:**

We conducted a two-year field experiment in five forests along the western edge of the Sichuan Basin in China, where atmospheric N deposition was up to 82–114 kg N ha^–1^ in the study sites. Four levels of N treatments were applied: (1) control (no N added), (2) low-N (50 kg N ha^–1^ year^–1^), (3) medium-N (150 kg N ha^–1^ year^–1^), and (4) high-N (300 kg N ha^–1^ year^–1^), N additions ranging from 40% to 370% of ambient N deposition. The decomposition processes of ten types of forest litters were then studied. Nitrogen additions significantly decreased the decomposition rates of six types of forest litters. N additions decreased forest litter decomposition, and the mass of residual litter was closely correlated to residual lignin during the decomposition process over the study period. The inhibitory effect of N addition on litter decomposition can be primarily explained by the inhibition of lignin decomposition by exogenous inorganic N. The overall decomposition rate of ten investigated substrates exhibited a significant negative linear relationship with initial tissue C/N and lignin/N, and significant positive relationships with initial tissue K and N concentrations; these relationships exhibited linear and logarithmic curves, respectively.

**Conclusions/Significance:**

This study suggests that the expected progressive increases in N deposition may have a potential important impact on forest litter decomposition in the study area in the presence of high levels of ambient N deposition.

## Introduction

Combustion of fossil fuels, nitrogen (N) fertilizer use, cultivation of N_2_-fixing crops, and other human activities have substantially altered the global N cycle and greatly accelerated the formation and deposition of reactive forms of N [1], [2]. Alteration of the N cycle has also greatly affected the cycle of carbon (C) on a global scale [3]. In many N addition studies conducted in forest ecosystems, plant growth (C fixation) nearly always responds positively [4], whereas the effect of N addition on litter decomposition (C release) varies considerably [5]. Several studies have reported significantly lower rates of litter decomposition in the presence of N additions [6]–[9]. However, many others have reported either no significant change [10] or a stimulation of decomposition [9]–[12]. Several chemical, biological, and biochemical hypotheses have been proposed to attempt explain the underlying mechanisms of chronic N deposition on litter decomposition, but was not well understood [7], [13]–[18].

A meta-analysis study indicated that the ambient N deposition level, N addition rate, and litter quality are the most important factors determining how litter decomposition responds to N additions [19]. Although anthropogenic inputs of N are estimated to have doubled the amount of active N entering the global terrestrial environment annually, the spatial distribution of N deposition is very uneven [2]. In general, relatively undisturbed areas are currently receiving relatively low inputs of anthropogenically fixed N via atmospheric deposition. Conversely, in highly developed areas, inputs of fixed N may actually be several times higher than in preindustrial times [20]. Furthermore, it has been predicted that over the next few decades, the greatest increments in anthropogenic N deposition will occur in areas that currently witness high ambient N deposition rates [2].

However, most N addition studies have been conducted in forest ecosystems with relative low ambient N deposition rates [19]. The effects of N addition on forest litter decomposition, in ecosystems receiving increasingly higher levels of ambient N deposition each year, are poorly understood. Southern China is one of the regions experiencing the most intensive N deposition activity [21]–[25], and significant increase in N deposition is expected to occur over the next few decades [26]. To evaluate the effect of N deposition on forest litter decomposition on the western edge of the Sichuan Basin (one of the most important industrial-agricultural economic regions in China), we performed experimental N additions in five different forests in this area over a two-year period. We tested the hypothesis: even under high ambient N deposition rates, experimental (anthropogenic) N addition would have a significant negative effect on forest litter decomposition because of potential inhibition effects of exogenous inorganic N on lignin decay.

## Materials and Methods

### Site Description

The study was conducted in five different forest sites, in Sichuan Province, China, near the western edge of the Sichuan Basin; the sites were named according to their respective dominant tree species (Table 1). This area experiences a moist subtropical highland climate [27]. Two of the sites were located in Yucheng County, the sites were approximately 500 m apart. The remaining three sites were located within an area of 1 km^2^ in Hongya County, 30 km away from Yucheng County. The *Pleioblastus amurus* (PA) site has been used for conducting studies on carbon sequestration [28] and soil respiration [29] under N addition experiments, and on N distribution and cycling through hydrological processes [30]. All the three research sites in this study are owned by Sichuan Agricultural University. The field studies did not involve endangered or protected species and no specific permits were required for the described field studies.

**Table 1:** Initial ecosystem characteristics at the experimental sites.

Site abbreviation	Dominant species	Family	Location	Elevation (m)	Aspect	Slope	Stand age (year)	Diameter at breast height (cm)	Stem density (stem ha^–1^)	Canopy density (−)	Soil pH(−)	Soil organic C (g kg^–1^)	Soil total N (g kg^–1^)
BD	*Bambusa pervariabilis* × *Dendrocalamopsis daii*	Gramineae, Bambusoideae	Hongya, 29°42′25′′N, 103°14′33′′E	600	W	5°	8	6	13320	0.9	4.2±0.4	13.4±0.2	1.58±0.02
BL	*Betula luminifera*	Betulaceae	Hongya, 29°42′25′′N, 103°14′38′′E	650	W	10°	8	15	1100	0.7	4.8±0.2	9.2±1.5	1.54±0.21
EG	*Eucalyptus grandis*	Myrtaceae	Yucheng, 29°58′48′′N, 102°58′58′′E	645	E	5°	6	20	1333	0.7	4.7±0.2	14.5±0.2	1.25±0.1
NA	*Neosinocalamus affinis*	Gramineae, Bambusoideae	Yucheng, 29°58′38′′N, 102°59′25′′E	670	N	6°	18	5.9	7500	0.9	4.6±0.01	11.4±0.2	0.94±0.1
PA	*Pleioblastus amarus*	Gramineae, Bambusoideae	Hongya, 29°42′25′′N, 103°14′13′′E	585	N	4°	8	2.3	52000	0.9	4.6±0.1	8.9±0.2	0.81±0.01

Soil properties were measured based on 0–10 cm, 0–10 cm, 0–20 cm, 0–5 cm, and 0–20 cm soil horizon in BD, BL, EG, NA, and PA, respectively. Values of soil pH, soil organic C, and soil total N are expressed as mean±s.d. (*n* = 3).

Values are expressed as mean ± s.d. (*n* = 3).

A 40 m×40 m plot was established at each site in October 2007 and subdivided into twelve sub-plots, measuring 3 m×3 m each, at about 5 m intervals. The twelve sub-plots were randomly allocated to four treatments: control (no N added), low-N (50 kg N ha^–1 ^year^–1^), medium-N (150 kg N ha^–1 ^year^–1^), and high-N (300 kg N ha^–1 ^year^–1^), with three replicates for each treatment. Ammonium nitrate (NH_4_NO_3_) was used as the N source and was applied monthly from November 2007 through October 2008.

### Nitrogen Deposition Assays

Atmospheric N deposition in the Yucheng and Hongya counties was measured in the open clearings adjacent to the BL and NA sites, respectively. Continuous 10-min precipitation intensity data was recorded using a Vantage Pro Weather Station (Davis Inc., Hayward CA, USA). In addition, event precipitation samples were collected, using six trough collectors with an area of 0.2 m^2^ each, from January 1, 2008 to December 31, 2010. The collectors, installed on supports 1 m above the ground, were constructed using PVC pipes (1 m long and 200 mm in diameter); these were connected via flexible tubes to 40-L plastic canisters. Each collector was covered by a 1-mm nylon mesh (cleaned regularly) to prevent plant debris, insects and other materials from entering the collectors. Precipitation samples were collected after each event (or next morning for events by day ending after 09∶00 pm local time) for the measurement of dissolved N concentrations. The sample collectors were rinsed with deionized water following each collection. All samples were collected in Nalgene polyethylene bottles that had been pre-washed with 5% HCl and rinsed thoroughly with deionized water. Samples were transported on ice, filtered through polycarbonate membranes (0.45 µm, Whatman Corp., Pittsburgh, UAS), and stored at –20°C until analysis. Total dissolved N was measured using a total C & N analyzer (TOC-V_cPH+TNM-1_, Shimadzu Inc., Kyoto, Japan); NH_4_^+^ was determined by colorimetry using Nessler’s reagent [31]; NO_3_^–^ was measured by colorimetry using a UV-1102 spectrophotometer (Tianmei Inc., Beijing, China) at 220 and 275 nm [31]. Organic N was calculated by the difference between total N and the sum of NH_4_^+^-N and NO_3_^–^-N.

### Experimental Treatments

Freshly fallen litter (Table 2) was collected on nylon mesh screens and sorted into one to three litter fraction(s): leaves, sheaths, and twigs, during June 2007 at each site. Next, each mesh litter bag (20 cm×20 cm, 1 mm mesh size) was filled with 10.00 g of air-dried litter for each fraction, sewn shut, and placed on the litter layer surface in their own forest. Decomposing litter in the bags was collected at 2- to 4-month intervals for 2 years (for 1 year except for the BL site) for each litter fraction from each plot. The litter materials were air-dried, removed from the litterbags, and gently separated adhering extraneous materials. Samples were then oven dried at 65°C for 48 h and weighed. Individual samples were ground using a Wiley mill with a 1-mm mesh screen and stored in paper bags for subsequent analyses.

**Table 2:** Initial chemistry of the investigated litter substrates.

Litter substrate	C (g kg^–1^)	N (g kg^–1^)	P (g kg^–1^)	K (g kg^–1^)	Ca (g kg^–1^)	Mg (g kg^–1^)	Lignin (g kg^–1^)	Cellulose (g kg^–1^)	C/N	N/P	Lignin/N
Lv_BD	374±2	13.10±0.03	1.06±0.01	3.66±0.13	2.23±0.05	14.13±0.12	111±2	245±2	29±0	12.3±0.0	8.5±0.2
Lv_BL	440±7	9.11±0.10	0.27±0.01	7.15±0.25	0.58±0.01	1.90±0.09	337±4	241±9	48±1	33.7±0.5	37.0±1.5
Lv_EG	452±6	12.06±0.25	0.65±0.03	4.64±0.24	0.35±0.09	1.75±0.02	211±2	152±6	37±1	18.6±1.1	17.5±0.4
Lv_NA	456±13	22.28±0.44	1.75±0.03	3.75±0.11	2.28±0.34	25.28±0.99	200±5	236±8	20±0	12.7±0.8	9.0±0.3
Lv_PA	389±6	3.88±0.10	0.35±0.01	2.75±0.12	1.15±0.15	2.68±0.10	208±10	135±5	100±1	11.2±0.0	53.8±3.5
St_NA	514±5	1.94±0.15	0.23±0.01	2.10±0.04	0.88±0.01	5.39±0.75	184±8	265±0	265±0	8.4±0.1	108±5.2
St_PA	465±6	5.19±0.05	0.11±0.01	3.61±0.11	0.17±0.02	0.16±0.02	190±12	212±12	90±0	46.7±3.0	36.6±2.0
Tg_EG	468±7	2.47±0.05	0.23±0.02	1.22±0.09	0.21±0.02	0.86±0.15	243±4	201±10	189±3	10.7±0.3	98.4±4.2
Tg_NA	493±4	1.57±0.03	1.11±0.15	1.66±0.08	0.59±0.01	6.13±0.12	225±11	115±1	115±1	3.8±0.1	127±6.5
Tg_PA	482±6	3.53±0.03	0.18±0.01	3.84±0.20	0.04±0.00	0.35±0.03	180±2	324±6	137±2	19.4±1.4	51.0±0.1

Values are expressed as mean ± s.d. (*n* = 9).

Lv_BD, Lv_BL, Lv_EG, Lv_NA and Lv_PA indicate leaf litter at the BD, BL, EG, NA and PA sites, respectively; St_NA and St_PA indicate sheath litter at the NA and PA sites, respectively; Tg_EG, Tg_NA and Tg_PA indicate twig litter at the EG, NA and PA sites, respectively.

### Chemical Analyses

Lignin concentrations were determined using the acid detergent fiber (ADF) method [32]. Briefly, ADF was prepared by refluxing 1.0-g of air-dried sample in acidified cetyltrimethyl ammonium bromide (CTAB) solution using a raw fiber extractor (FIWE, VELP, Milan, Italy). Suspensions were filtered through a Gooch-type crucible (40–50 µm, VELP, Milan, Italy) rinsed three times with boiling water, and then rinsed twice with cold acetone, to yield colorless and transparent suspensions. The residual materials were acidified with 72% (w/v) sulfuric acid (H_2_SO_4_) for 3 h. The cellulose concentration was determined based on mass loss following this acidification of the ADF. Reaction residues were again filtered through a Gooch-type crucible (40–50 µm, VELP, Milan, Italy) and rinsed with boiling water, until the suspensions were colorless and transparent. They were then dried to a constant mass in an oven (130°C) and their weights recorded as *W*_1_. The lignin concentration was calculated as the difference between *W*_1_ and the residual mass (*W*_2_) of the filtrate after ignition in a muffle at 550°C for 2 h (i.e., *W*_1_– *W*_2_). Total N concentration in the litter was determined through acid digestion, using the Kjeldahl method [33]. Briefly, a 200-mg sample was digested in 5 mL of 1.84 g mL^–1^ (18.4 M L^–1^) H_2_SO_4_ and then distilled using a UDK 142 automatic distillation unit (VELP, Milan, Italy). For the determination of P, samples were subjected to triple-acid digestion (nitric, perchloric, and sulphuric acid, 5∶1∶1, v/v/v) [34]. Total P was determined colorimentrically in digested samples using the ammonium molybdate/stannous chloride method [35]. The concentrations of K, Ca, and Mg were analyzed using an atomic absorption spectrophotometer (TAS-986, PGENERAL, Beijing, China) following perchloric acid-nitric acid (HClO_4_-HNO_3_) digestion [36]. Their concentrations were expressed per unit of oven dried sample (65°C).

### Statistical Analyses

The residual substrate mass, as a proportion of the initial mass, was plotted against time, using a single-exponent decomposition model, *X* = *e* ^–*kt*^ [37], where *X* is the fraction of initial mass remaining at time *t*, and *k* is the decomposition constant. To investigate the effects of N addition on decomposition, we compared *k* among treatments using a one-way analysis of variance (ANOVA) for each substrate (SPSS v15, SPSS Inc. Chicago, USA). The relationships among initial chemical parameters and the *k*-values were fitted using linear and nonlinear regression models.

## Results

### Nitrogen Deposition

The average annual precipitation in Yucheng and Hongya was 1958 and 1822 mm, respectively, from 2008 to 2010. The mean wet N deposition in Yucheng and Hongya was 94 and 95 kg N ha^–1^ year^–1^, respectively, during the same period (Table 3).

**Table 3:** Mean annual precipitation (mm) and wet N deposition rates (kg N ha^–1^ year^–1^) in Yucheng and Hongya counties, Sichuan Province, China.

Year	Yucheng		Hongya	
	Precipitation	wet N deposition	Precipitation	wet N deposition
2008	1666±18	97±7	1758±10	82±7
2009	1922±22	93±8	1984±6	114±9
2010	2286±19	93±7	1724±15	90±8
Mean	1958±20	94±6	1822±19	95±8

Values are expressed as mean ± s.d. (*n* = 6).

### Litter Mass Loss and k value

**Figure 1:**
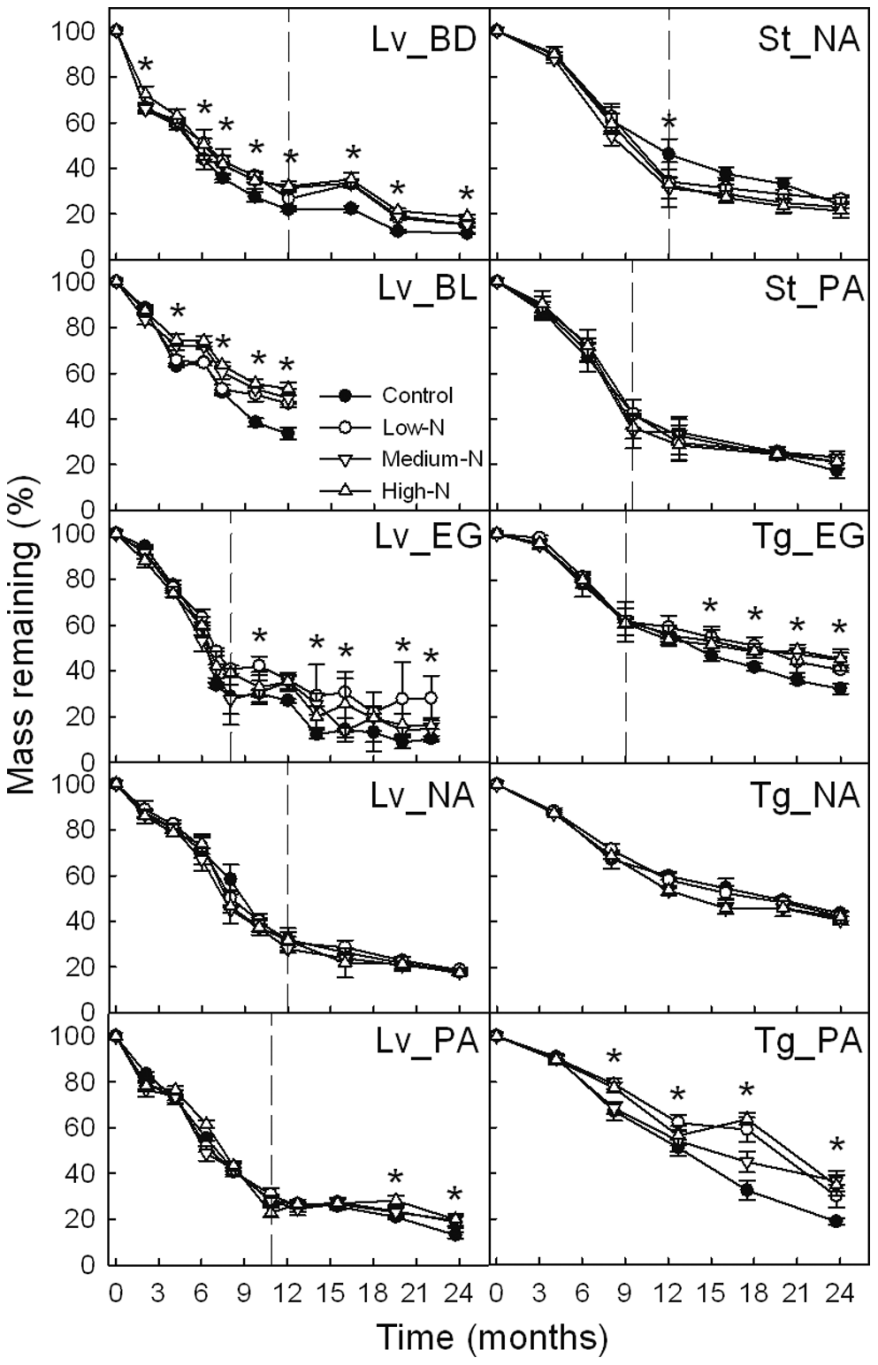
Percentages of substrate mass remaining in each of the different N treatments. Error bars represent the standard deviations of the means (*n* = 3). Lv_BD, Lv_BL, Lv_EG, Lv_NA and Lv_PA indicate leaf litter at the BD, BL, EG, NA and PA sites, respectively; St_NA and St_PA indicate sheath litter at the NA and PA sites, respectively; Tg_EG, Tg_NA and Tg_PA indicate twig litter at the EG, NA and PA sites, respectively. Asterisks (*) indicate significant difference between the control and at least one N treatment (*α* = 0.05). Two distinct stages were observed for most of the litter substrates (initial rapid decomposition rate followed by a slower rate) separated by dashed lines.

**Figure 2:**
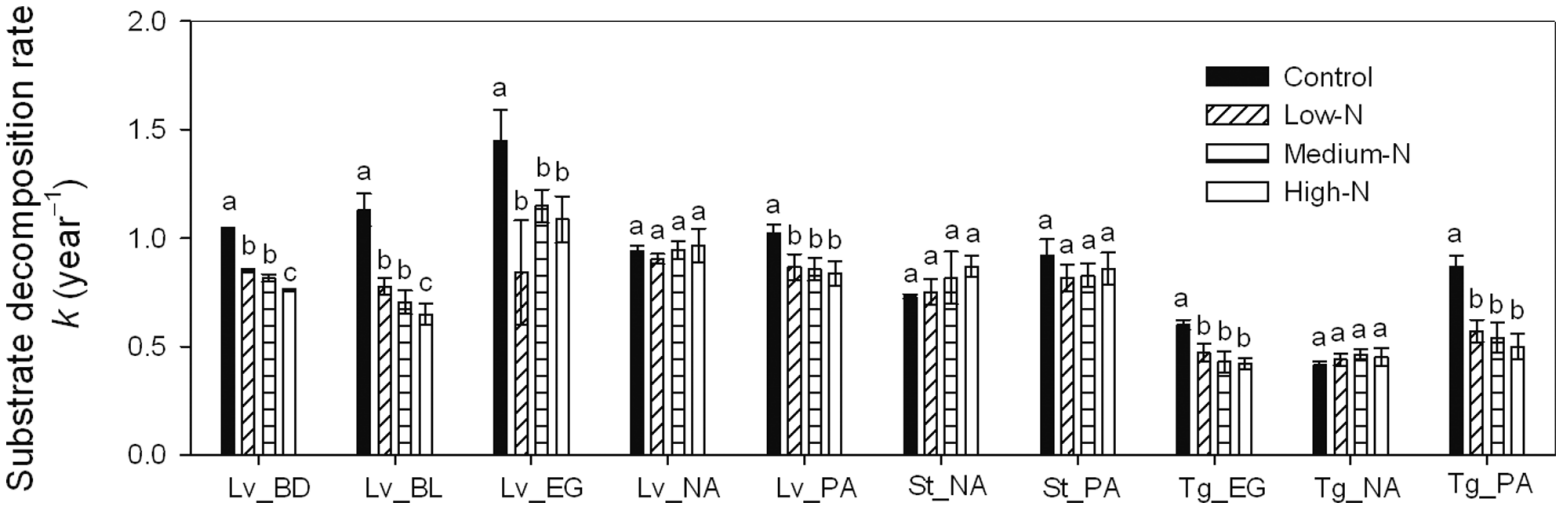
Annual decomposition rates (*k*) of substrates in the control and N-addition plots. Lv_BD, Lv_BL, Lv_EG, Lv_NA and Lv_PA indicate leaf litter at the BD, BL, EG, NA and PA sites, respectively; St_NA and St_PA indicate sheath litter at the NA and PA sites, respectively; Tg_EG, Tg_NA and Tg_PA indicate twig litter at the EG, NA and PA sites, respectively. Error bars represent the standard deviations of the means (*n* = 3). Different letters denote significant differences in *k* values among N treatments within each substrate (one-way ANOVA, *n* = 3).

The single-exponent decomposition model was a good fit for plotting the fraction of initial mass remaining in each litter substrate and treatment as a function of time (*P*<0.001) (Fig. 1). Two evident stages of decomposition were observed with respect to most of the litter substrates investigated, namely, a rapid rate of decomposition early, followed by a slower rate in later stages. After two years of decomposition, the mass losses of leaves, sheaths and twigs ranged from 76% to 91% (mass loss of leaf litter at BL was 49%–69% in one year), 71% to 99%, and 54% to 81%, respectively. Nitrogen addition significantly decreased the *k*-values of six of the ten substrates (four leaf litters and two twig litters), but had no significant effect on the *k*-values of the other four substrates (Fig. 2).

**Figure 3:**
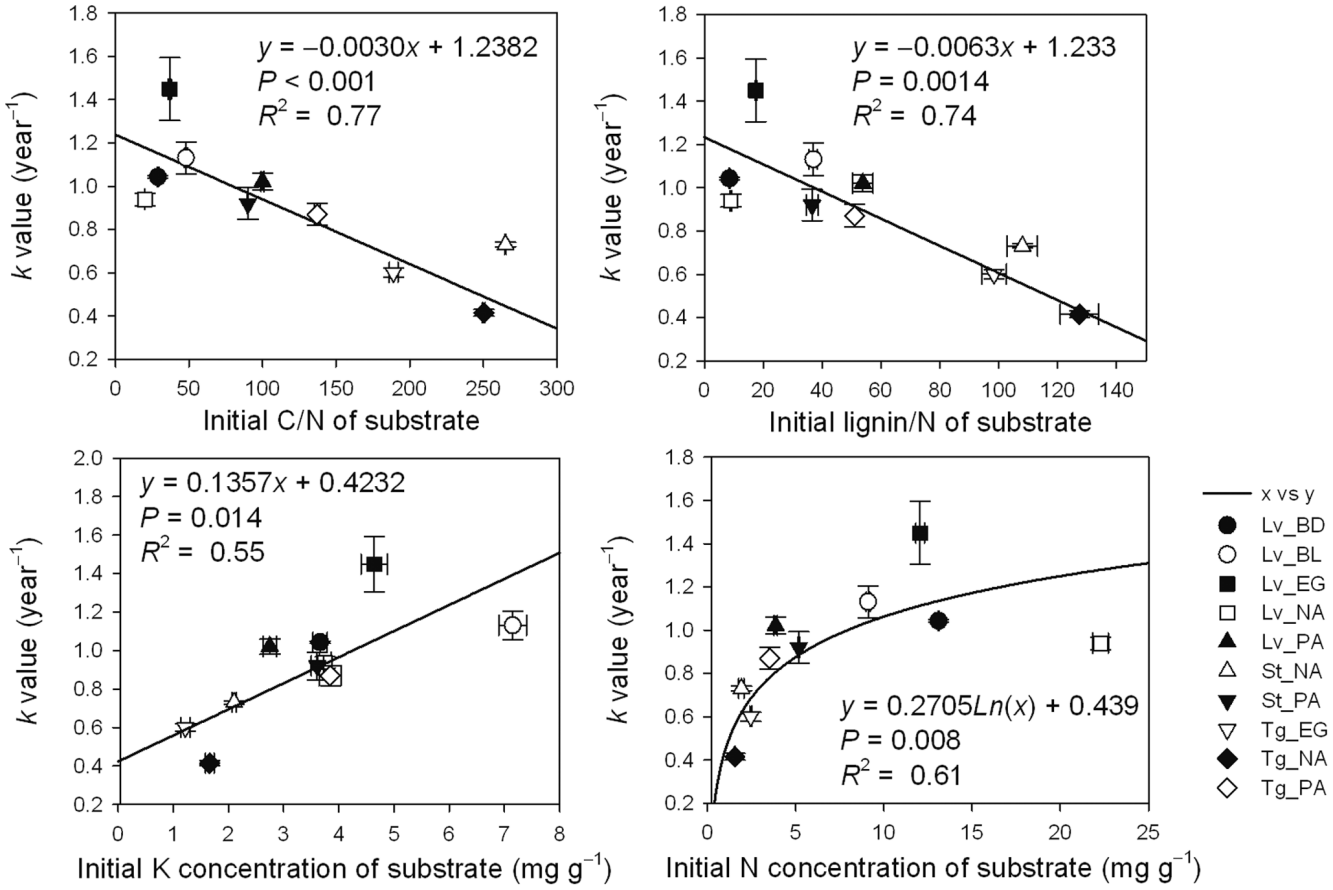
Relationships between the of initial chemical properties of substrates and their annual decomposition rates (*k*). Lv_BD, Lv_BL, Lv_EG, Lv_NA and Lv_PA indicate leaf litter at the BD, BL, EG, NA and PA sites, respectively; St_NA and St_PA indicate sheath litter at the NA and PA sites, respectively; Tg_EG, Tg_NA and Tg_PA indicate twig litter at the EG, NA and PA sites, respectively. Error bars represent the standard deviations of the means (*n* = 3).

The relationships of *k*-values to initial C/N and to lignin/N were assessed using several models, including linear, exponential and logarithmic models. We found significant negative linear relationships of *k*-values to initial C/N, and to Lignin/N, but significant positive linear relationships between *k*-values and initial K concentration, positive logarithmic relationships between *k*-values and initial N concentration (Fig. 3).

### Lignin and Nitrogen

**Figure 4:**
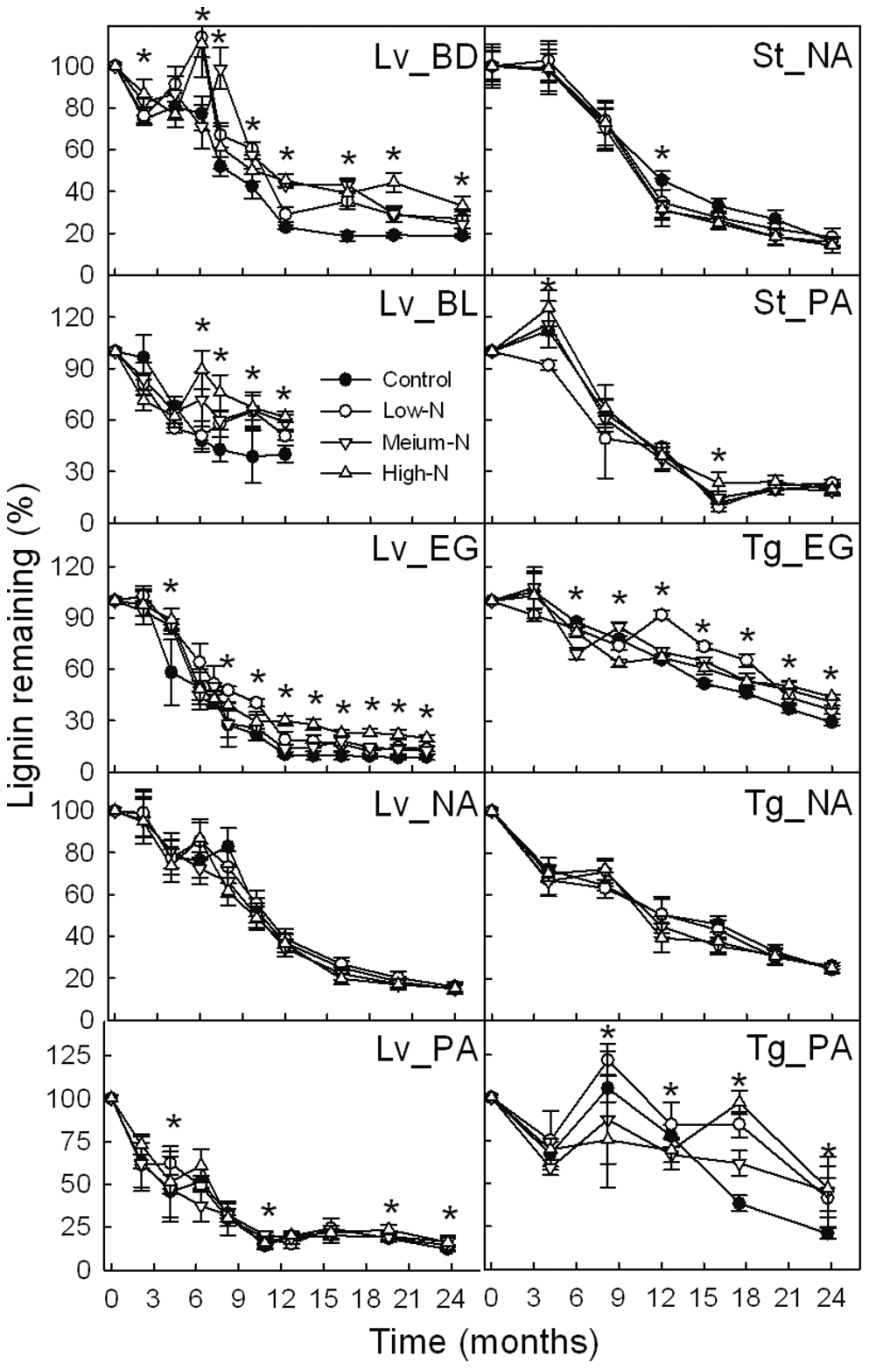
Percentage of residual lignin in each substrate in different N treatments. Lv_BD, Lv_BL, Lv_EG, Lv_NA and Lv_PA indicate leaf litter at the BD, BL, EG, NA and PA sites, respectively; St_NA, St_PA indicate sheath litter at the NA and PA sites, respectively; Tg_EG, Tg_NA and Tg_PA indicate twig litter at the EG, NA and PA sites, respectively. Error bars represent the standard deviations of the means (*n* = 3). Asterisks (*) indicates significant differences between the control and at least one N treatment (*α* = 0.05).

**Figure 5:**
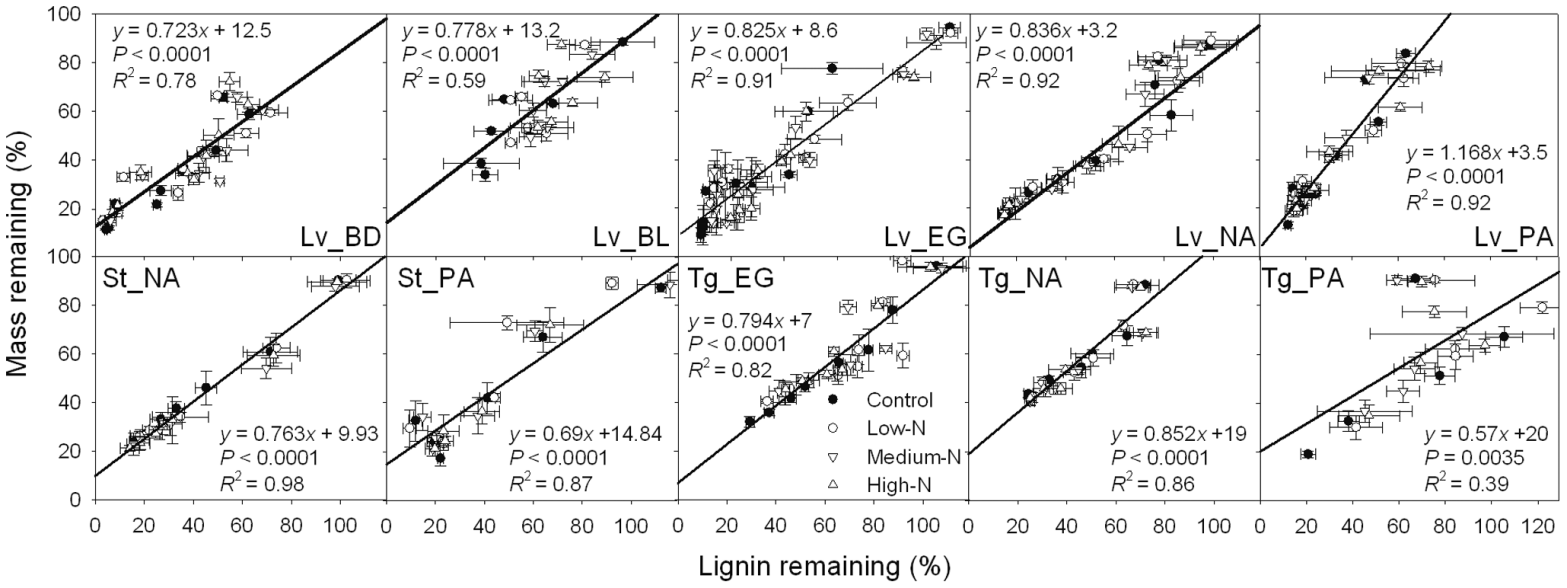
Relationships between residual lignin and substrate mass after a period of decomposition. Lv_BD, Lv_BL, Lv_EG, Lv_NA and Lv_PA indicate leaf litter at the BD, BL, EG, NA and PA sites, respectively; St_NA and St_PA indicate sheath litter at the NA and PA sites, respectively; Tg_EG, Tg_NA and Tg_PA indicate twig litter at the EG, NA and PA sites, respectively. Error bars represent the standard deviations of the means (*n* = 3).

Similar to the mass loss, decomposition of lignin was depressed by N addition in later stages of the same substrates as their *k*-value affected by N addition (Fig. 4). Nitrogen addition had no significant effect on the amount of lignin remaining of the other four substrates. Further, there were significant positive linear relationships between mass remaining and lignin remaining, for all of the ten substrates (*P*<0.01, *R* ^2^>0.39) (Fig. 5).

**Figure 6:**
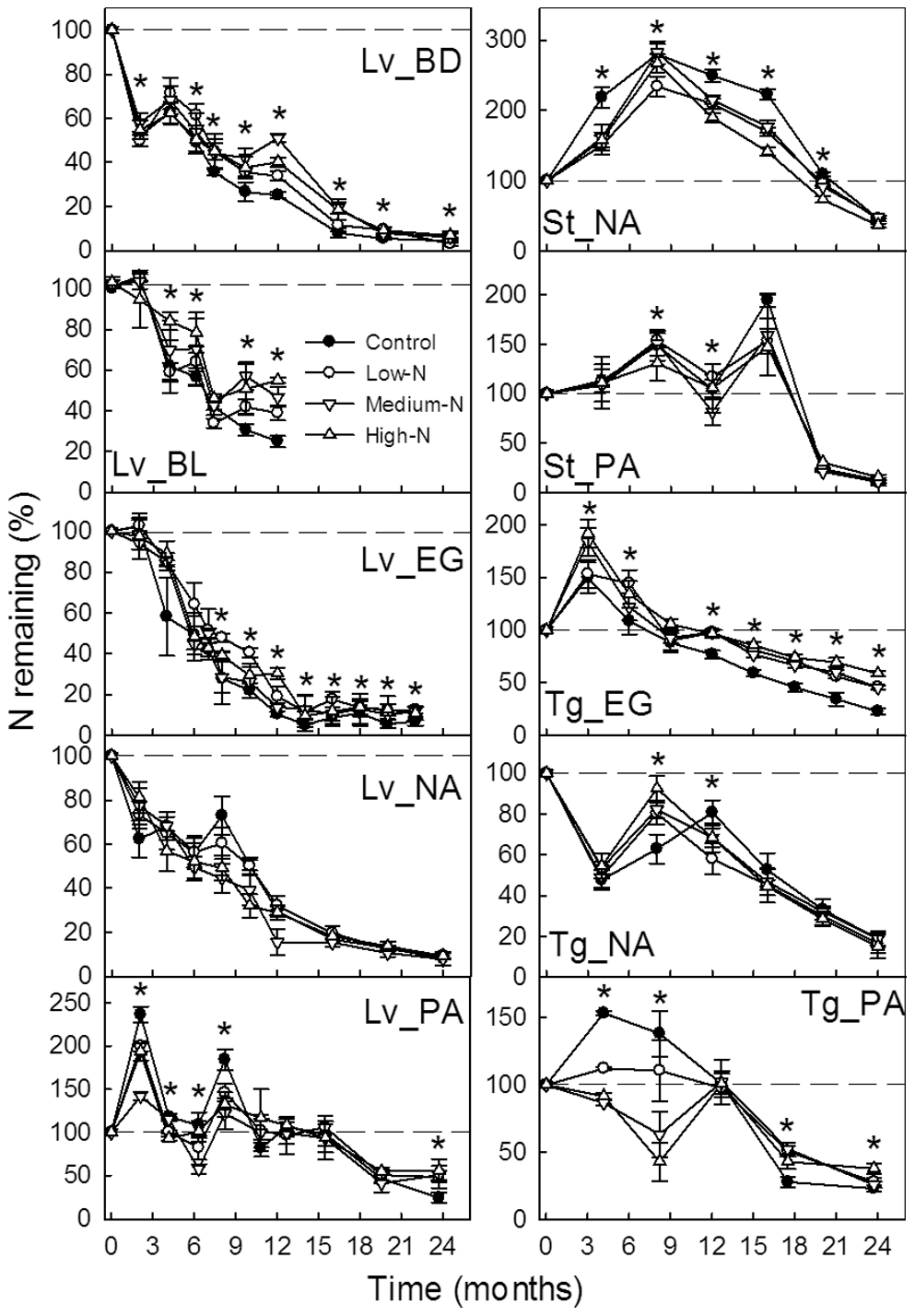
Percentage of nitrogen remaining in each substrate in different N treatments. Lv_BD, Lv_BL, Lv_EG, Lv_NA and Lv_PA indicate leaf litter at the BD, BL, EG, NA and PA sites, respectively; St_NA and St_PA indicate sheath litter at the NA and PA sites, respectively; Tg_EG, Tg_NA and Tg_PA indicate twig litter at the EG, NA and PA sites, respectively. Error bars represent the standard deviations of the means (*n* = 3). Asterisks (*) indicates significant difference between the control and at least one N treatment (*α* = 0.05).

Nitrogen dynamics differed among substrates (Fig. 6). Six substrates (Lv_PA, St_NA, St_PA, Tg_EG, Tg_NA and Tg_PA) with initial N concentrations lower than 5.5 g N kg^–1^ exhibited a net immobilization of N during the early stages of decomposition. On the other hand, net N mobilization occurred at the initiation of decomposition of the other four substrates with initial N concentrations ranging from 9–22 g kg^–1^. In general, all of the substrates released N in the later stage of decomposition. During this stage, N addition significantly increased the amount of residual N in the six substrates but had no effect on residual N in the other four substrates.

## Discussion

### Atmospheric Nitrogen Deposition

High rates of wet N deposition were observed at the investigated sites (82–114 kg N ha^–1^ year^–1^, mean of 95 kg N ha^–1^ year^–1^), which is significantly higher than the average N deposition rate across China (15.8 kg N ha^–1^ year^–1^ [38]) and the average N deposition rate that was reported across 50 forest sites in China (16.6 kg N ha^–1^ year^–1^ [24]). In addition, this is exponentially higher than the N deposition rates observed in the US (3.0 kg N ha^–1^ year^–1^ [39]) and Europe (6.8 kg N ha^–1^ year^–1^ [39]). To our knowledge, the N deposition rates observed in our study are among the highest reported worldwide [40]. There are several possible reasons for this phenomenon. First, the Sichuan Basin is one of the most economically important industrial–agricultural regions in southwestern China. Rapid development in this region has resulted in significant increases in the emission of reactive N forms over the last few decades. Second, reactive N released from across Sichuan Basin and from the municipality of Chongqing (located near the eastern edge of the basin) may have been transported specifically to the western edge of the basin by monsoonal winds. Orographic lift contributes significantly to high precipitation rates (1500–2000 mm annually) in the elevated western side of the Sichuan Basin (known as the “rainy zone” of western China [27]).

### Effect of Nitrogen Addition on Litter Decomposition

Confirming our hypothesis, experimental N additions had a significant effect on forest litter decomposition, even under high ambient N deposition rates. In a meta-analysis of previous studies, Knorr et al. [19] reported that ambient N deposition and N fertilization rates were the key factors affecting litter decomposition. In this meta-analysis, the N fertilization rate generally ranged from 26 to 600 kg N ha^–1^ year^–1^, but ambient N deposition rates only ranged from <1 to 19 kg N ha^–1^ year^–1^. The authors hypothesized that N additions had no significant effect on litter decomposition in areas subject to chronically high levels of ambient N deposition. This is in stark contrast to the results reported in our study, where the decomposition rates of the six substrates were decreased significantly by N additions, particularly in the later stages of decomposition. These six substrates comprised four leaf litters and two twig litters. Leaf litter comprised the majority of above-ground litter input in these five ecosystems [41]; in our study, N additions inhibited the decomposition of most leaf litter substrates. Therefore, although atmospheric N deposition ranged from 82 to as much as 114 kg N ha^–1^ year^–1^, N additions of 40% to 370% of this amount still had significant effects on forest litter decomposition.

Inhibitory effects of N addition on lignin decomposition were observed in all the six substrates, whose mass losses were inhibited by N addition. In the later stages of litter decomposition, the relative concentration of lignin increased in the six substrates, which consistent with the hypothesis that exogenous inorganic N suppressed litter decomposition through inhibiting lignin decay. The residual mass of substrate was closely related to the amount of residual lignin, which suggests that lignin may have a critical role in influencing litter mass loss. Nitrogen addition suppressed the decomposition of lignin in later stages, which in turn slowed the rate of mass loss. Many previous studies reported that exogenous inorganic N inhibits the decomposition of lignin [6], [14], [15], and then the mass loss of litter [6]–[8]. The inhibitory effects of N addition on lignin decomposition in the present study can be interpreted as N addition reduced lignolytic enzyme production and decomposer efficiency. In a previous study at the same sites, we reported that N additions significantly depressed the activities of polyphenol oxidase and peroxidase (the main lignin-decomposing enzymes) in the surface soil horizon (0–20 cm) [42]. Keyser et al. [43] found that white rot fungi were unable to synthesize lignin-decomposing enzymes when ammonia and amino acids accumulated. In the later stages of litter decomposition, fungal communities are limited by high lignin and N concentrations [43], resulting in a greater accumulation of humus in the surface layer of the forest soil [6], especially in forests with high-lignin litter [44].

The decomposition rate (*k*-value) was closely related to the initial chemical properties of the litter substrates. The *k*-value exhibited a significantly negative linear relationship with initial C/N and lignin/N, and significantly positive linear and logarithmic relationships with initial tissue K and N concentrations, respectively. This is in agreement with a recent meta-analysis that reported that litter quality parameters, such as the C/N ratio, control litter decomposition rates [45]. Several studies have reported that climate is the major factor affecting litter decomposition on a global scale [46], [47]. However, other studies have indicated that litter substrate quality, such as C/N, lignin/N, and total nutrient content, are the dominant factors controlling litter decomposition rates, even at the global scale [18], [45], [48]. Thus, litter substrate quality is one of the best predictors of *k*-values within a particular climatic region [46]. However, the implications of the positive logarithmic relationship between *k*-values and K content remain unclear.

In general, nutrient dynamics in decomposing litter are controlled by stoichiometric constraints [47], [49], [50]. Parton et al. [47] conducted a 10-year study of litter decomposition in 21 sites located over seven biomes, with the finding that net N release from litter was driven primarily by the initial tissue N concentration. They also reported that net N release occurred only when the average C/N ratio of the litter was less than 40. On the other hand, based on a 6-year study of litter decomposition in several biomes in Canada, Moore et al. [49] reported a C/N threshold of 55 for net N release. Our study paralleled this conclusion, based on analysis of the decomposition rates of the different litter substrates (leaves, sheaths, twigs) in the five investigated subtropical. The C/N ratios of four of the substrates (leaf litter at the BD, BL, EG and NA sites) were lower than 50, while, the C/N ratios of the other six substrates were greater than 90. Therefore, we inferred that net N release occurred at the initiation of decomposition of the four substrates with relatively low C/N ratios, and continued until the end of the study period. On the contrary, net N immobilization occurred at the initiation of decomposition of the other, high-C/N substrates. This echoes the findings of our previous study in two subtropical bamboo forests, where we reported that the patterns and dynamics of N, P, K, Ca and Mg contents, and their residual contents after litter decomposition, were primarily determined by initial substrate quality [41].

### Potential Effects of Nitrogen Deposition on Carbon Status

Litter decomposition is the first step in soil organic matter formation and plays a key role in the flux of CO_2_ from the soil to the atmosphere [51], as well as in nutrient cycling and productivity in most terrestrial ecosystems [52]. Our findings in the current study indicated that the response of litter decomposition to N addition was generally negative at all five sites. However, total ecosystem C storage and C cycling depend on the balance between decomposition and production, they both can change under the projected increases in N deposition. Our observations have found that nitrogen additions for two years enhanced carbon sequestration in a bamboo forest (PA site) through the stimulation of plant growth [28]. Aboveground litter production in this forest was increased by N additions, as a consequence of increased plant biomass [53]. The increased litter input and the inhibitory effect of external N on litter decomposition were expected to increase the soil C pool in the PA stand [28]. However, the indirect effects (such as on litter quality and subsequent litter decomposition) of N additions may be compensatory. For example, at these sites, we found that N addition significantly increased N concentrations in leaf litter [52]. A similar phenomenon has been reported in other studies as well [38], [54]. Therefore, the indirect effects of N addition on leaf litter quality will likely manifest as an increase in the decomposition rate in the early stages [5].

Overall, the present study suggests that the mean N deposition rate along the western edge of the Sichuan Basin is higher than the mean N deposition in mainland China [25]. Despite the high rates of ambient N deposition, N additions decreased forest litter decomposition by inhibiting the decomposition of lignin. This study suggests that the increase in N deposition rates may have a potentially significant impact on the C cycle of forest ecosystems, assuming that ambient N deposition continues to increase as predicted. However, the results from this two-year experiment cannot necessarily be extrapolated to predict the long-term status of litter decomposition or even C cycles in such ecosystems. More long-term fixed sample site studies among different biomes are needed in the future to supplement our understanding regarding N deposition on global C cycle.
